# Feasibility study of portable multi-energy computed tomography with photon-counting detector for preclinical and clinical applications

**DOI:** 10.1038/s41598-021-02210-5

**Published:** 2021-11-23

**Authors:** Chang-Lae Lee, Key Jo Hong, Namwoo Kim, Kwanhee Han, Dongkyu Kim, Hoe-Su Jung, Sangmin Lee, Junyoung Park, Kyoung-Yong Lee, Jee Eun Lee, Yuna Choi, Minkook Cho

**Affiliations:** 1 PCD R&D Group, Health & Medical Equipment Business Unit, Samsung Electronics, Suwon-si, Gyeonggido Korea; 2 Laboratory Animal Center, Daegu Gyeongbuk Medical Innovation Foundation, Daegu, Korea; 3 Department of Family Medicine, Bupyeong Serim Hospital, Incheon, Korea

**Keywords:** Cancer, Medical research, Optics and photonics

## Abstract

In this study, preclinical experiments were performed with an in-house developed prototypal photon-counting detector computed tomography (PCD CT) system. The performance of the system was compared with the conventional energy-integrating detector (EID)-based CT, concerning the basic image quality biomarkers and the respective capacities for material separation. The pre- and the post-contrast axial images of a canine brain captured by the PCD CT and EID CT systems were found to be visually similar. Multi-energy images were acquired using the PCD CT system, and machine learning-based material decomposition was performed to segment the white and gray matters for the first time in soft tissue segmentation. Furthermore, to accommodate clinical applications that require high resolution acquisitions, a small, native, high-resolution (HR) detector was implemented on the PCD CT system, and its performance was evaluated based on animal experiments. The HR acquisition mode improved the spatial resolution and delineation of the fine structures in the canine’s nasal turbinates compared to the standard mode. Clinical applications that rely on high-spatial resolution expectedly will also benefit from this resolution-enhancing function. The results demonstrate the potential impact on the brain tissue segmentation, improved detection of the liver tumors, and capacity to reconstruct high-resolution images both preclinically and clinically.

## Introduction

Compared to conventional energy-integrating detector (EID)-based computed tomography (CT), photon-counting detector CT (PCD CT) provides significant advantages such as reduced electronic noise, increased contrast-to-noise ratio (CNR), decreased metal artifacts, improved spatial resolution, material decomposition, and dose efficiency^1–6^. The primary difference between the two detectors is that EID integrates all photon energy levels while PCD discriminates between the energy levels of each incident photon by counting the number of photons according to a defined energy threshold. The functionality of the PCD CT system along with its many advantages for preclinical and clinical purposes have been verified in many reports published earlier^7–9^.

Treatment of ischemic stroke is critically dependent on the time after the incident, and an early diagnosis is thus paramount. Magnetic resonance imaging (MRI) is helpful for the detection of early signs of ischemic stroke and for the determination of treatment strategies in the acute phase^10,11^. Typically, native CT scans are performed first to exclude the possibility of intracranial haemorrhage. If the haemorrhage is absent, CT perfusion imaging by using iodinated contrast agents and/or CT angiography are conducted to detect the ischemic stroke blockage region in the blood vessel. While a CT system is preferred owing to its speed and widespread availability, brain tissue segmentation and diagnosis of the acute phase of ischemic stroke are more difficult compared with MRI. However, there are ongoing studies that aim to detect early signs of the ischemic stroke by using segmentation of the white and gray matters and tissue-specific thresholds^12–14^.

As every substance has a unique X-ray attenuation and energy dependence pertaining to the attenuation coefficients, characterization of the X-ray energy distribution transmitted to the PCD can help to distinguish decomposed substances among tissues and the contrast agents^8,15,16^. In this study, the brain tissue was segmented by applying machine learning techniques to multi-energy brain images acquired by using the PCD CT based on the characterization of energy distribution in the tissue. Brain images were acquired by using a 3 T MRI scanner to allow a comparison of the segmented white and gray matter images.

Early diagnosis of hepatocellular carcinoma (HCC) is critical in improving the prognosis of the patient^17^. Although the detection rate of the cancer nodules has increased with the development of CT hepatic arteriography (CBCT-HA)^18,19^, small-sized hyper-vascular HCC are often missed or are poorly detected in the early diagnosis made by using CT imaging. In addition, HCC with atypical enhancement patterns and those with sizes ≤ 1 cm have low-detection rates^20^. In this study, we quantitatively evaluate iodine-enhanced tumors distributed in rabbit liver after isolation.

In our previous study, we evaluated the energy resolution, detector efficiency, ability of a CdTe detector and its iodine quantification accuracy, by capitalizing on the clinical potential of the PCD CT system in generating the reduced metal artifacts in head phantom CT scans^21^. As the next logical step that should succeed the previously performed phantom experiment and the performance evaluation study of the detector, this study demonstrates the clinical imaging scenarios in the brain, liver, aorta of two different animal models─the canine (head region) and the rabbit (liver with tumor, aorta). Furthermore, to accommodate the clinical applications, e.g., for CT imaging of the nasal region that requires high resolution, the spatial resolution was improved by using a small, native, high-resolution (HR) detector implemented on the PCD CT system and evaluated based on animal experiments.

Herein, we report our investigation of the potential impact of PCD CT system on brain tissue segmentation, improved detection of liver tumors, and demonstrate its capacity to generate high-resolution images preclinically and clinically.

## Methods

### Photon-counting detector module

An in-house pixelated CdTe detector prototype was developed using an application-specific integrated circuit (ASIC) that had 3840 parallel readout channels. A CdTe layer of 1.4 mm thickness was prepared. A pixelated anode (size 230 μm × 190 μm) and a common electrode at the cathode were coated on each side of the prepared CdTe layer. The thickness of the anode and cathode was 0.1 μm and 0.2 μm, respectively. Each ASIC readout channel was connected to one CdTe pixel. All the channels were equipped with a charge-sensitive preamplifier, three discriminators, digital-to-analog converter (DAC), and three counters. The bit-depth of the three counters for low-, middle-, and high-energy detections were 14, 13, and 12, respectively. The preamplifier had a feedback circuit to compensate for the leakage current. To decrease the power consumption, the discriminators operated in the current mode. Additionally, the 6-bit DAC was included for compensation offset in each pixel. The detector assembly included 48 detector modules. Each detector module had an array size of 80 × 48 pixels. This means that the detector assembly had 80 rows and 2,304 columns which allowed the collection of 80 simultaneous slices of data with each rotation of the gantry. We have previously described the performance of the CdTe detector in terms of the energy resolution, count rate (detector efficiency), and detector stability^18^.

### Spectral CT system design

**Figure 1:**
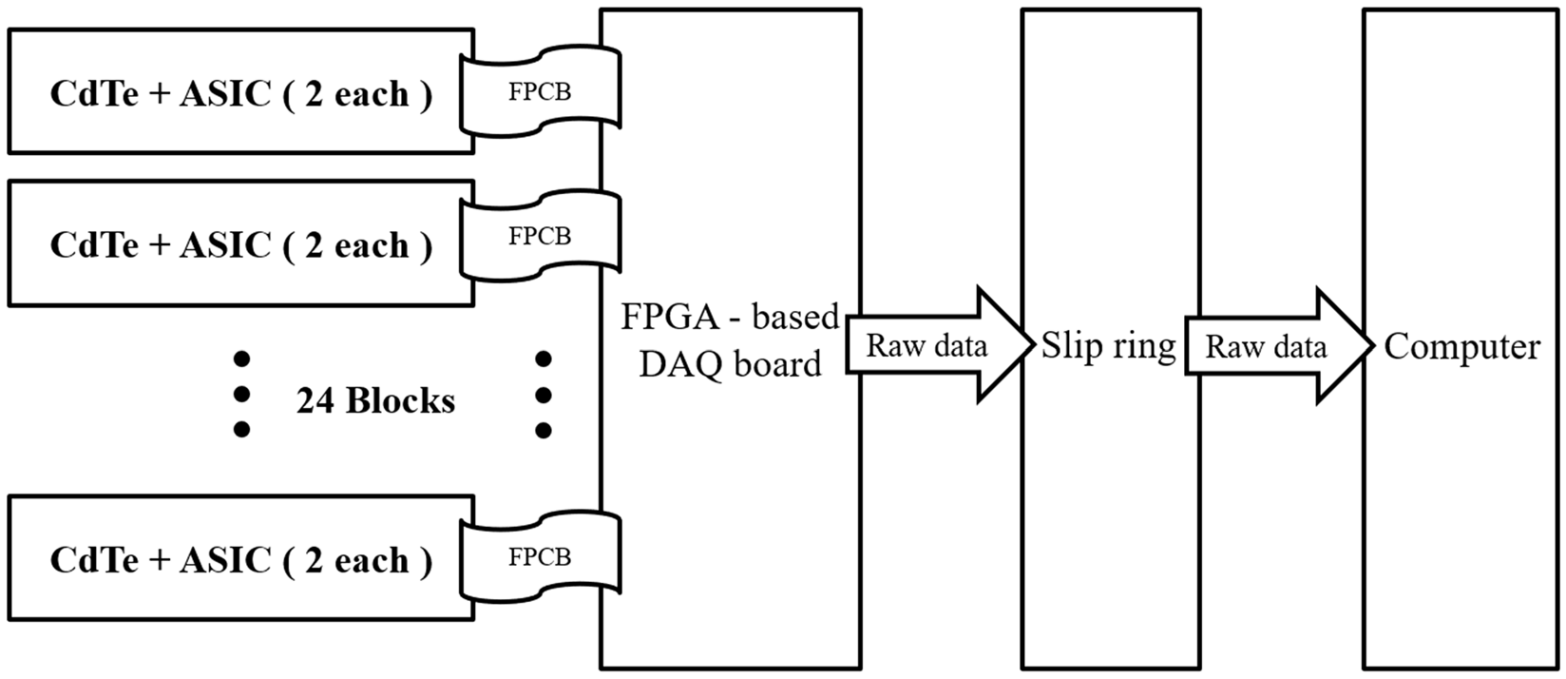
Photon-counting detector system consisting of CdTe, application-specific integrated circuit (ASIC), and various other components.

**Figure 2:**
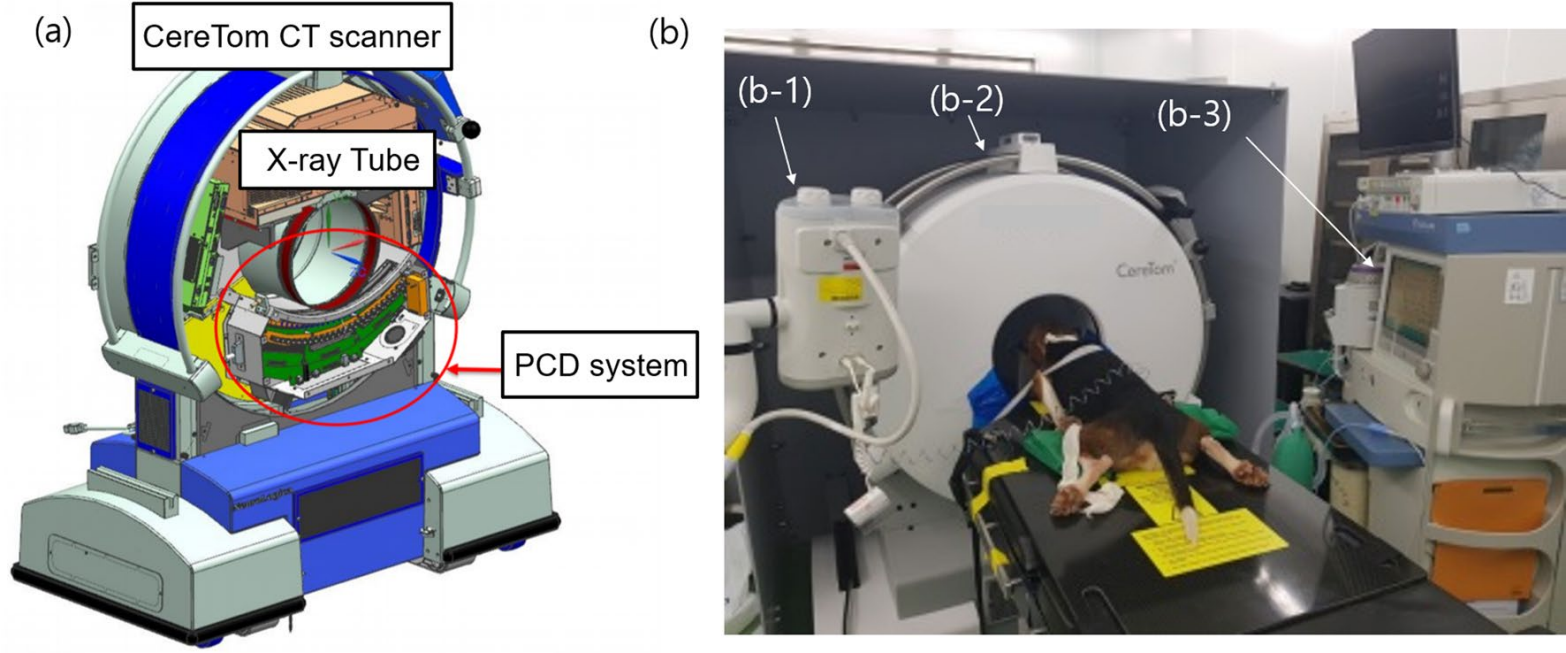
(**a**) Illustration of the photon-counting detector computer tomography (PCD CT) system placed inside the CereTom Scanner. (**b**) Beagle dog positioned for scanning. (b-1) CT injection system for injecting contrast medium. (**b-2**) PCD CT system. (**b-3**) Monitoring system for temperature, blood pressure, peripheral oxygen saturation, and end-tidal carbon dioxide.

As shown in Fig. 1, the CT system has 48 detector modules arranged in a curved frame with a source-to-isocenter distance of 227.5 mm. The axial field-of-view (FoV) of the CT system is 250 mm. Each detector block consists of two PCD modules and a flexible printed circuit board (FPCB). The photon count signals generated from the CdTe with the ASIC are transmitted through the FPCB to the data acquisition (DAQ) units. The ASIC digital signals are sent to a custom-made DAQ unit with a field programmable gate array (FPGA)-based board. The digital signals are processed by the DAQ unit, which has 10 FPGAs and 12 GB of memory. The signal data containing the energy information from the three energy bins are stored in the memory and are subsequently transferred to a hard disk in a computer. The prototypal photon-counting detector modules were designed and fabricated with appropriate physical dimensions to specifically fit a portable CereTom CT scanner (Samsung Neurologica, Boston, Massachusetts, USA)^22^. The conventional EID modules of the CereTom scanner were replaced with the PCD system^18^. Figure 2a shows an illustration of the PCD system location. The PCD counts the X-ray photons transmitted through the patient and sorts the counts in the three energy bins. The three bins of count data are processed to produce three sets of uncorrected raw data (low-, middle-, and high-energy), which are used as inputs to the CT reconstruction algorithm.

### Animal preparation

#### Beagle dog preparation

Two healthy male beagle dogs (Orient Bio, Gyeonggi, Korea), weighing 11–13 kg were used for this experiment. The dogs were kept on fasting overnight before they were anaesthetised. First, they were subcutaneously injected with 0.05 mg/kg atropine sulfate (Daihan Pharm, Seoul, Korea). After 10 min, the dogs were sedated with 2 mg/kg xylazine (Bayer, ON, Canada) which was administered intramuscularly. Anaesthesia was induced after 10 min by 2 mg/kg alfaxalone (Jurox, NSW, Australia) administered as a slow intravenous injection, and maintained with 1–2% isoflurane mixed with 1.0 L/min 100% O_2_ after intubation. Temperature (37–39 °C), pulse (80–120 beats/min), sPO_2_ (> 95%), and end-tidal CO_2_ (ETCO_2_) (40–50 mmHg) were monitored during the CT scan by using a Primus Anesthesia machine (Dräger, Lübeck, Germany) and an Infinity Kappa patient monitor (Dräger, Lübeck, Germany).

#### VX2 tumor model preparation

Seven New Zealand white male rabbits (Samtako, Gyeonggi, Korea), weighing 2.1–2.5 kg were used for induction of VX2 liver carcinoma^23^. Donor rabbits (n = 2) were used to propagate the VX2 tumor (provided from Utah-Inha DDS & Advanced Therapeutics Research Center). Anaesthesia was induced by 5 mg/kg alfaxalone (Jurox, NSW, Australia) injected intramuscularly and maintained with 1–2% isoflurane mixed with 1.0 L/min 100% O_2_. With the use of an 18-gauge needle, 1.0 mL of VX2 cell solution was injected into the hindlimb muscle. After three weeks, the donor rabbits were sacrificed. Immediately thereafter, the VX2 tumor was excised from their hindlimbs. The surrounding connective tissue, fat, and necrotic region were cleaned from the tumor. The tumor was then sectioned into 1 mm^3^ cubes in phosphate buffer solution (PBS) solution and was kept on ice. These small tumor cubes were inoculated in the anterior lobe of the liver of five recipient rabbits after they were anaesthetised with 5 mg/kg alfaxalone injected intramuscularly (Jurox, NSW, Australia) and 1.5–2% isoflurane (Hana Pharm, Korea) was administered via inhalation. Gentle pressure was applied using sterile cotton swabs on the inoculation site in the livers to prevent the tumor cubes from coming out. The tumors were then left to grow for three weeks. All rabbits were anaesthetised by intramuscular injection of zoletil (15 mg/kg) and xylazine (10 mg/kg), and sacrificed by administering KCL intravenous (IV) injection under anaesthetised condition by deep inhalation of Isofluran > 5%.

### Image acquisition

The current PCD CT system with total energy range of 30–140 keV and the three energy bins (bin 1: 30–50 keV, bin 2: 50–65 keV, and bin 3: 65–140 keV) can acquire four multi-energy projections from a single scan with an arbitrary keV setting. The total energy of the bins are the combined energy of bins 1, 2, and 3. Energy calibration was performed by using two additional filters: gadolinium (K-edge energy = 50.2 keV) and tungsten (K-edge energy = 69.5 keV) to set the bins for all energy levels^21^. Images were acquired in a 5 × 6 binning (standard) mode, with a the slice thickness 0.640 mm at the isocenter, and 16 slices per scan. Axial scans were acquired with a voltage of 140 kV, currents in the range of 1–7 mA, gantry rotation time of 2 s, and 1,440 projections per rotation. Images were reconstructed with the use of a filtered back-projection (FBP) algorithm and a FoV of 250 mm.

#### Beagle dog head images

The beagle dog head images acquired to evaluate the basic image quality of the PCD CT system are shown in Fig. 2b. Pre- and post-contrast axial CT images of the canine’s brain were acquired within the total energy range and at the various multi-energy bins. The head scan was performed in an axial scan mode with a tube voltage of 140 kV, a tube current of 5 mA, and a speed of 2 s per gantry rotation. For contrast-enhanced imaging, the dogs were administered with 2 mL/kg of iodinated contrast media (Iomeron 350 mg/mL; Bracco, Milan, Italy) via a 20 G intravenous catheter (injection rate of 2 ml/s) placed in the cephalic vein by using a power injector (MEDRAD Stellant CT Injection System, Medrad, City, PA, USA). Instead of the PCD CT system, by adapting a gantry rotation time of 2 s, an EID-based Biograph mCT 128 (Siemens Healthineers, Erlangen, Germany) was used to improve uniformity in the arterial enhancement and to perform a test bolus during the time the contrast agent took to reach the brain after the cephalic vein injection. All head scan images were acquired by keeping dogs in a sternal recumbent position. In addition, the Hounsfield unit (HU) values were compared based on images obtained from the canine’s head reconstructed from the PCD CT and EID CT systems. Because the system’s geometry and X-ray tube specifications were different in the two CT systems (PCD and EID), the volume CT dose index (CTDI_vol_) values of the two scans were matched to ensure that the same amount of radiation dose was used to image each section. The HU measurements were determined within a manually drawn region-of-interest (ROI) on the CT images with the use of ImageJ (version 1.45, NIH, Bethesda, MD, USA)^24^. Circular ROIs covering areas as large as possible within each of the tissues and materials were used. Dog brain images were acquired by using a 3 T MRI scanner (MAGNETOM Skyra, Siemens Healthineers, Erlangen, Germany) for comparing with CT images of the brain. A two-dimensional fluid-attenuated inversion recovery (FLAIR) sequence was used with the following parameters: repetition time (TR)/echo time (TE)/inversion time (TI) = 10,000/91/2500 ms, 130 × 130 mm FoV, 256 × 256 acquisition matrix, 180° flip angle, 2 mm slice thickness, and 2.5 mm slice gap.

#### Rabbit liver tumors

Before the CT scan, the location, size, and blood distribution of the tumors in the rabbit liver parenchyma were measured using Doppler ultrasonography. The doppler ultrasonography images were obtained by using an E-CUBE 15 ultrasound device (Alpinion Medical Systems, Gyeonggi, Korea) equipped with a liner L3-12X transducer (3–12 MHz). The rabbits were sedated before the imaging by an intramuscular injection of 5 mg/kg alfaxan (Jurox, NSW, Australia) for the induction and maintained with 1–2% isoflurane mixed with 1.0 L/min 100% O_2_. An 18-gauge catheter was placed in the ear vein of the rabbits to administer the contrast agent. Iodinated contrast agent (1.5 mL/kg, Iomeron 350 mg/mL, Bracco, Milan, Italy) was injected at a flow rate of 2 mL/s, and the hepatic arterial phase image was acquired 6 s after the administration of the contrast agent. An axial scan was performed within a range that could sufficiently cover the tumor in the liver parenchyma. The optimal contrast agent injection time and image acquisition time were assessed based on the preliminary experiments by using multiple image acquisitions to determine the maximum peak concentration of the contrast agent within the liver tumor region. Circular ROIs (tumor, normal liver parenchyma, and aorta) were defined manually as areas as large as possible within each of the evaluated tissue regions. These ROIs were placed in homogeneous regions of the structures of interest, and were then automatically copied onto the iodinated map. The reported data denote the mean ± standard deviation (SD) values. Contrast-to-noise ratios (CNRs) were calculated using the formula, CNR = (ROI_tissue_ – ROI_muscle_)/SD_noise_, where ROI_tissue_ is the mean value of the tumor, liver and aorta, ROI_muscle_ is the mean value measured in a circular or oval ROI drawn on the back muscle region, and SD_noise_ is the noise of the measured values. The ROIs of all the regions were measured thrice.

#### Evaluation of new HR imaging technology in PCD CT system

An HR mode was implemented on the PCD CT system of the research scanner to take advantage of the smaller native detector pixel size and to improve the spatial resolution to accommodate the clinical applications in the nasal regions and the tempotal bone. In the HR mode, 1 × 1 detector pixels were used instead of binning 5 × 6 pixels (standard mode). However, preliminary investigations of this HR mode took a long time (30 s) for data acquisition. Therefore, acquisition of the multi-energy bins was omitted, and data were acquired from within the total energy range, which is a single energy.

### Material decomposition using deep neural networks

**Figure 3:**
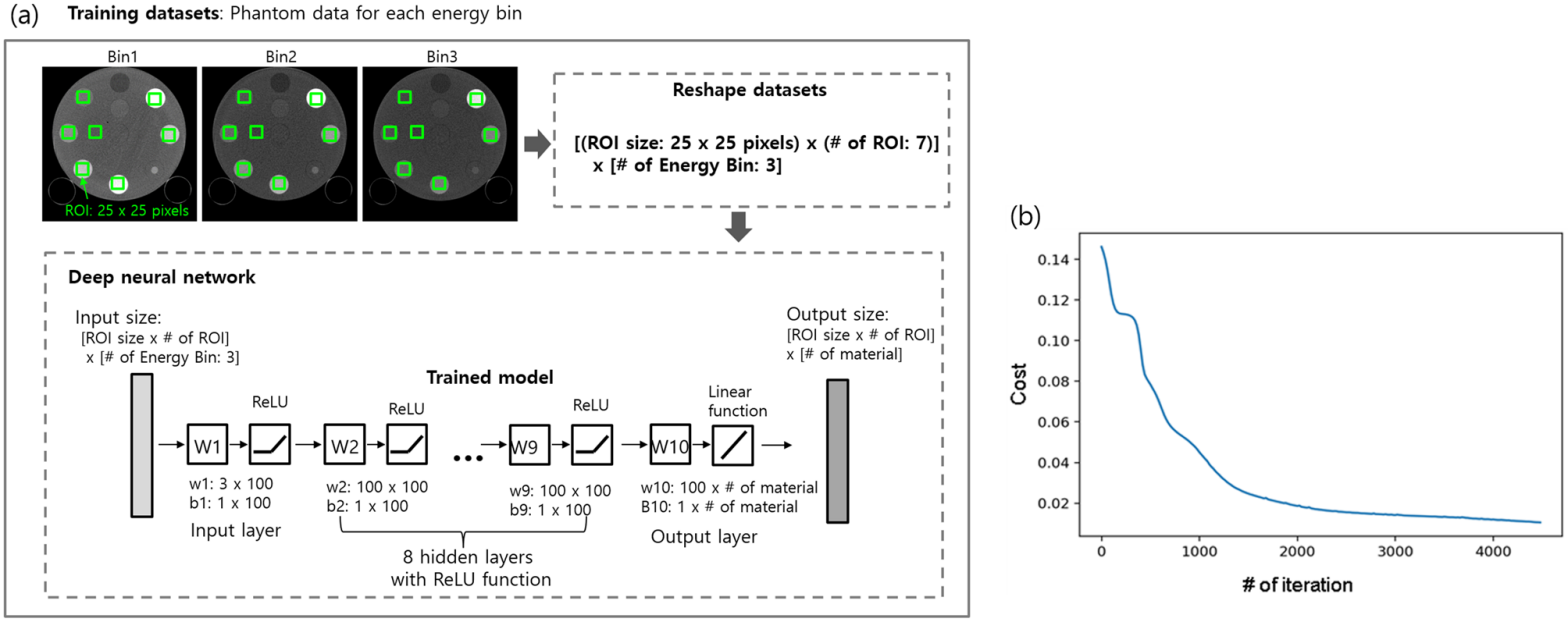
(**a**) Illustration of the structure for networks. The model consists of eight hidden layers with ReLU function. Images are shown as examples. (**b**) Cost function.

**Figure 4:**
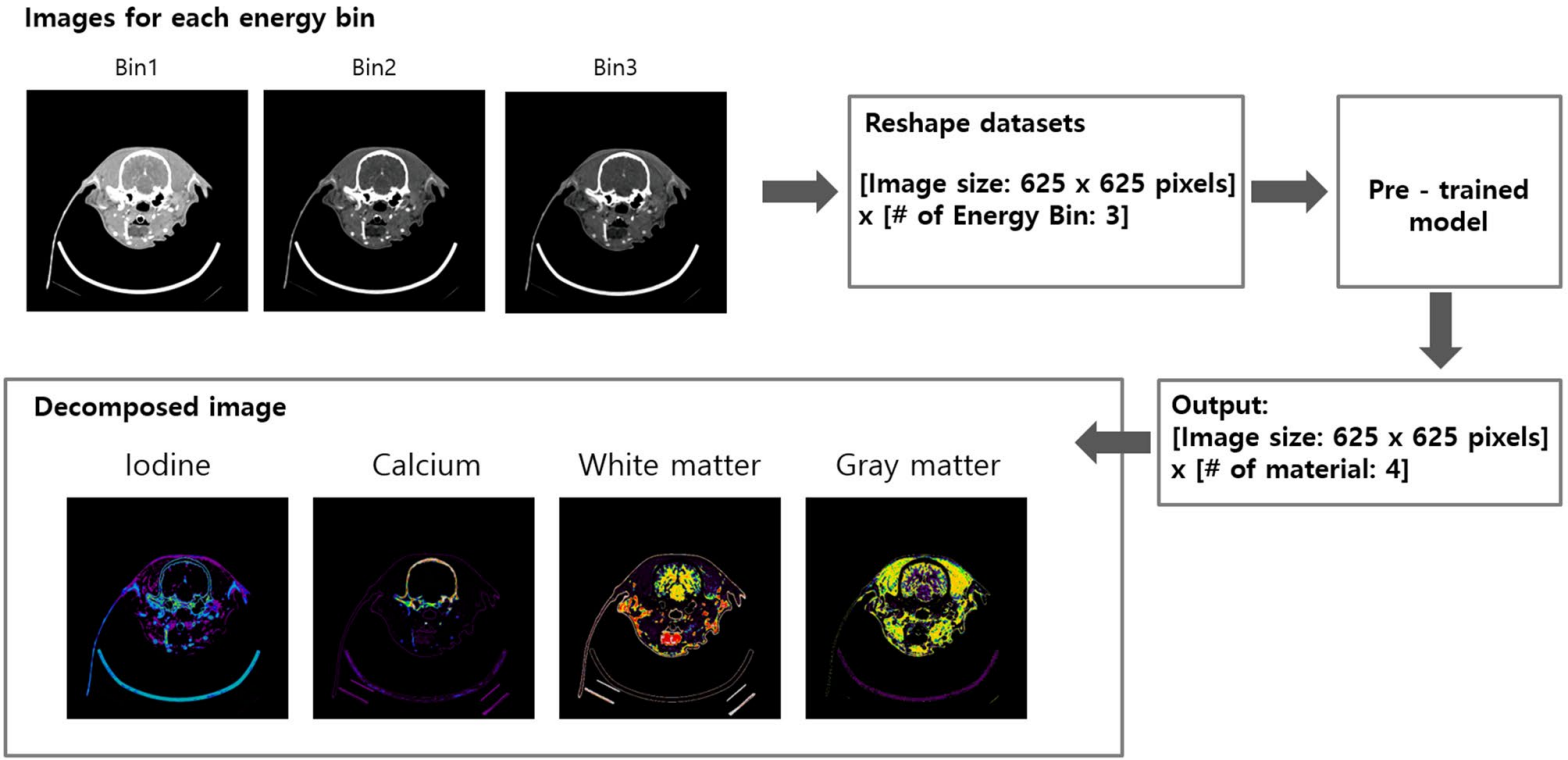
An example of application of the network for material decomposition.

In this study, we proposed a deep-learning-based material decomposition. Our method was performed in two steps: a training step using the multi-energy phantom (model 1472 Gammex) and the application of the pre-trained model to the test images. Figure 3a presents the network architecture. It was implemented using Python 3.6 with the Tensor Flow library. Figure 3b shows the cost function for training the model. For the training of the model, we used a multi-energy phantom with cylindrical holes and the real dog (for white and gray matter). The holes were filled with the iodine, calcium—at different concentrations—and water^21^. The projection images acquired from the multi-energy phantom were reconstructed for each energy bin. The ROI size was 25 × 25 pixels^2^. In the case of the multi-energy phantom and dog, seven and two ROIs were set for each energy bin respectively. Hence, the input size used for the training was (25 × 25 × 9) × 3 (energy bins). The model consisted of eight hidden layers with a ReLU function^25^. The output of the model was set to show the material-separated information for the input pixels. Figure 4 illustrates the material separation process performed by applying the model shown in Fig. 3. For example, if there is a reconstructed image in each energy bin, the image size of 625 × 625 pixels in each energy bin was reshaped in one dimension. With the reshaped image as the input, the obtained model weight was applied, as shown in Fig. 3. Upon doing so, an output of size 625 × 625 × (number of materials) was obtained. As shown in the example in Fig. 4, if the iodine, calcium, white matter, and gray matter were separated, a map for the four materials can be obtained by applying the model weight. When the training was conducted with a learning rate of 0.00005 for 4500 epochs, the cost function decreased and converged close to a value close to zero.

### Ethics statement

All animal experiments were performed according to the SOP(ex.SOP-ANI-10(03) for dog management, SOP-ANI-14(03) for rabbit management, SOP-ANI-21(01) administration method_dog, SOP-ANI-22(01) administration method_rabbit, SOP-ANI-34(03) environment enrichment, SOP-IACUC-03(01) Post-Approval Monitoring (PAM) after the IACUC approval. In addition, PAM was conducted to confirm that the experiment was performed following protocols as approved by the IACUC. All procedures used in the above animal experiment were approved by the Institutional Animal Care and Use Committee of Daegu-Gyeongbuk Medical Innovation Foundation in Republic of Korea (Permit Numbers: DGMIF-18071004-00 (dogs) and DGMIF-18051102-04 (rabbits)). All the experiments were performed in accordance with the relevant regulations and the ARRIVE guidelines.

## Results

### Multi-energy CT imaging

**Figure 5:**
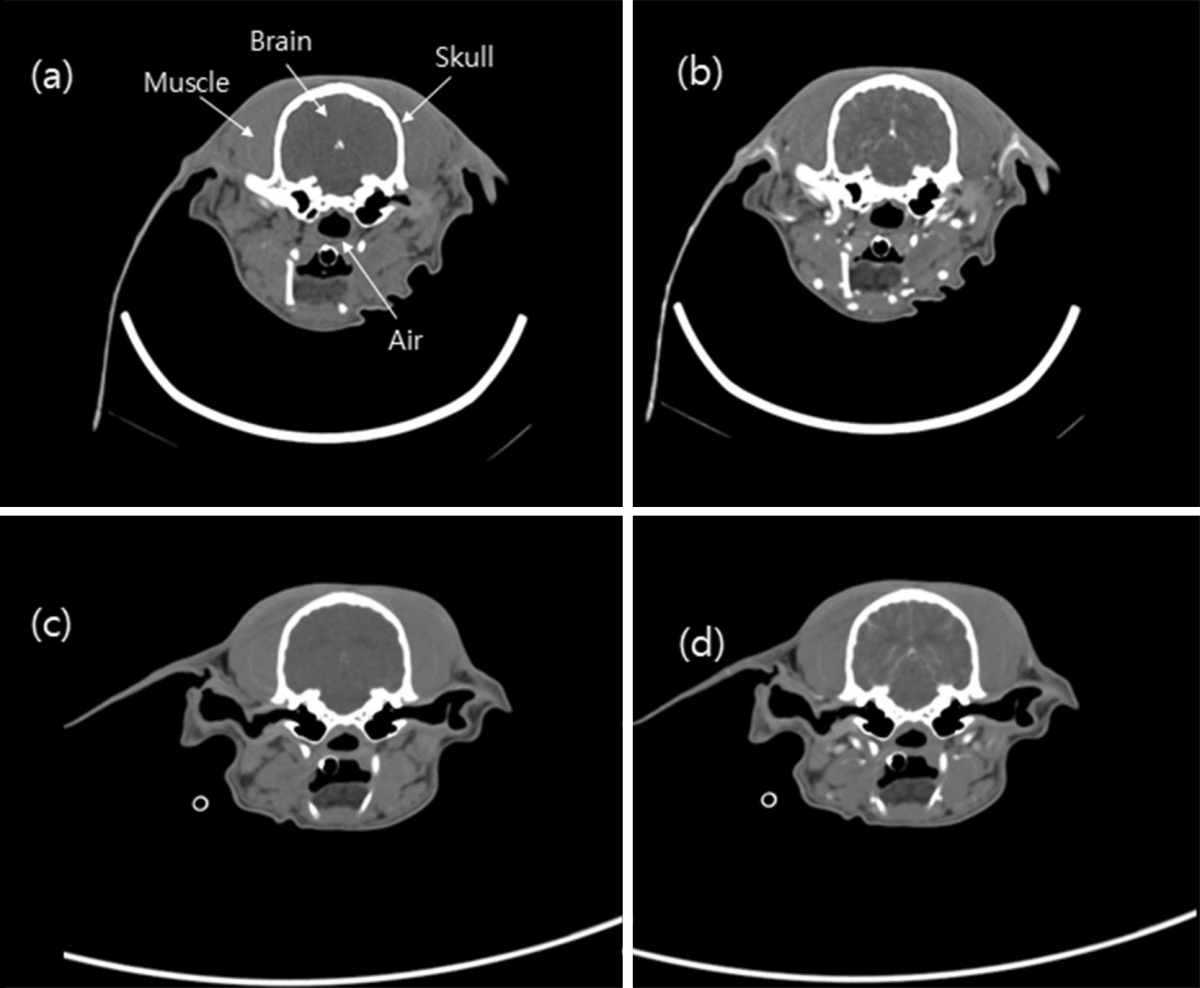
(**a**) Pre- and (**b**) post-contrast axial image of the canine’s brain in the PCD system (30–140 keV, 10 mAs, CTDIvol: 41.7 mGy, slice thickness: 2.5 mm, display window/level: W/L = 450/100 HU). (**c**) Pre- and (**d**) post-contrast axial image of the head in the EID system (140 kVp, 100 mAs, CTDIvol: 41.7 mGy, slice thickness: 2.0 mm, display window: W/L = 450/100 HU).

**Figure 6:**
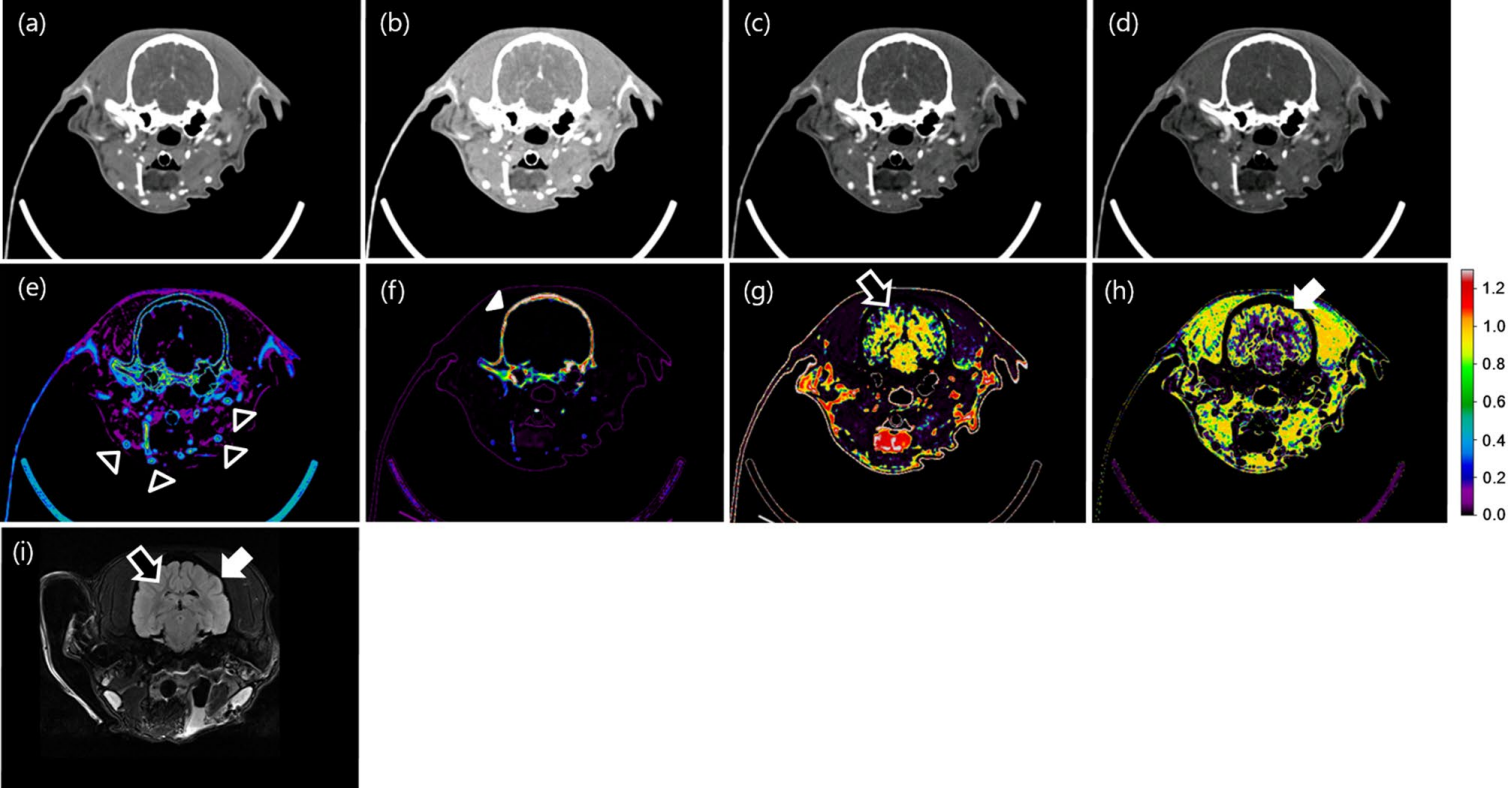
Contrast-enhanced axial images of the canine brain acquired at (**a**) energy levels in the range of 30 to 140 keV in the PCD CT system and (**b**) multi-energy bin 1: 30–50 keV, (**c**) bin 2: 50–65 keV, and (**d**) bin 3: 65–140 keV (CT dose index (CTDI_vol_): 41.7 mGy, display window: W/L = 450/100 HU, slice thickness: 2.5 mm). An image of iodine (**e**), calcium (**f**), white matter (**g**), and gray matter (**h**) separated with the use of images of the three energy levels (bins 1, 2, and 3). Axial brain MRI (**i**) of FLAIR sequence. Black arrowheads indicate arteries, the white arrowhead indicates the skull, the black arrow indicates white matter, and the white arrow indicates gray matter.

Figure 5 shows the pre- and post-contrast axial images of the canine’s brain obtained using the PCD and the EID CT systems. The mean HU values of the air, muscle, brain, and the skull images of the dog head in both the PCD CT system and the EID CT system were measured and compared. The difference in the mean HU values of the selected ROIs between the PCD and EID CT images was not significant. Figure 6a–d show the contrast-enhanced axial images reconstructed by the PCD CT system of the canine’s brain, acquired at the total energy range and the multi-energy bins. Additionally, images of the three energy levels (bins 1, 2 and 3) were used to generate the images of the separated iodine (Fig. 6e), calcium (Fig. 6f), white matter (Fig. 6g), and gray matter (Fig. 6h). Axial brain MRI acquired with the FLAIR sequence for comparison with the image of the PCD CT system is shown in Fig. 6i.

**Figure 7:**
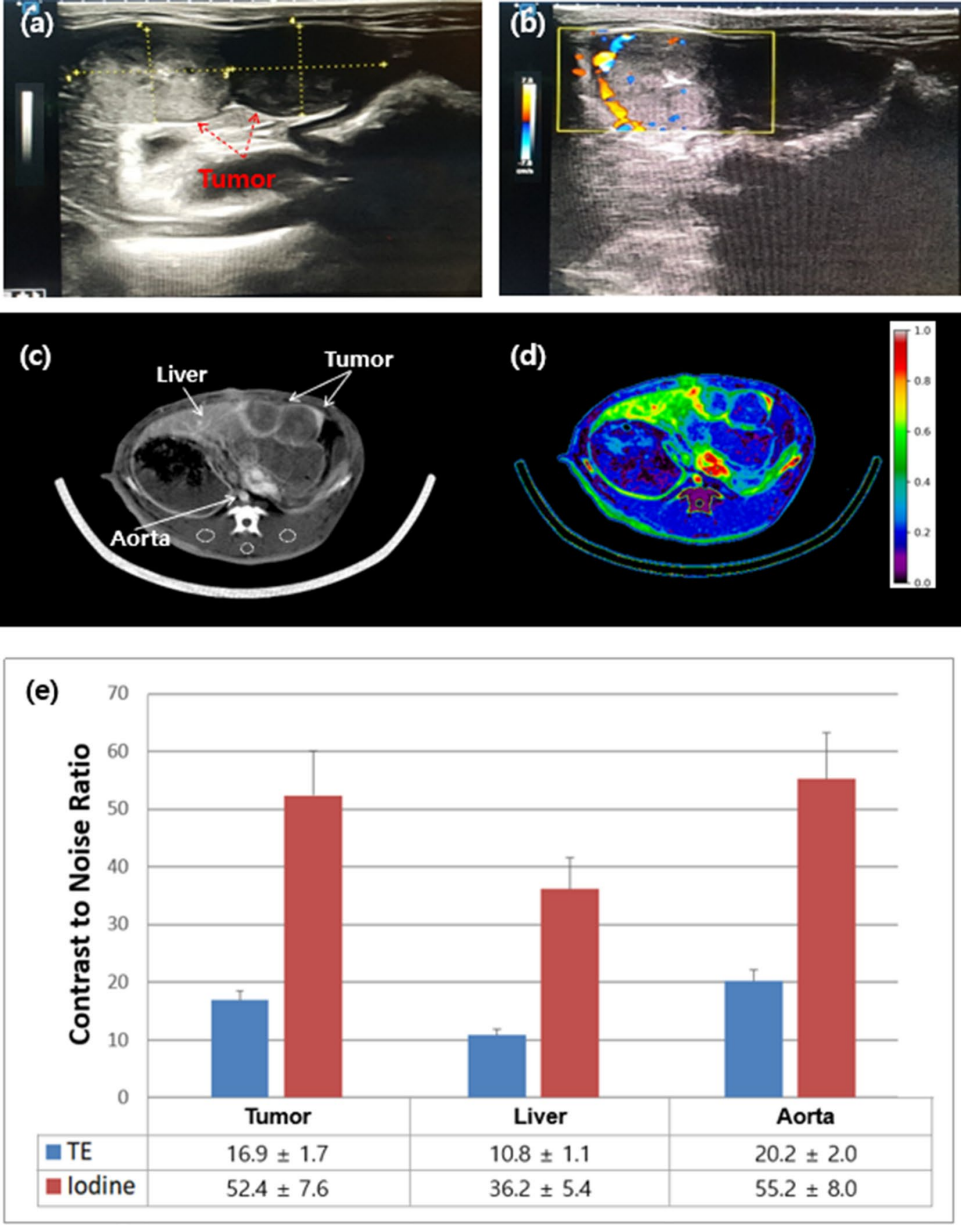
Rabbit with VX2 tumors located in the anterior part of the liver presented as (**a**) two-dimensional and (**b**) three-dimensional flow images produced via Doppler ultrasonography. (**c**) Contrast-enhanced axial image acquired at the total energy range and (**d**) iodine map image of the rabbit liver in the PCD CT system. The dotted circles are back muscles. 30–100 keV, 10 mAs, slice thickness = 2.5 mm, WW/WL [450/100]. (**e**) Contrast-to-noise ratio (mean ± SD) of the PCD CT system for the tumor, liver, and aorta in the total energy range (TE, 30–140 keV) of a rabbit acquired at an energy level in the range of 30–140 keV and the iodine map.

Ultrasonography was performed to assess the location and shape of the liver tumors (Fig. 7a), and Doppler imaging was used to identify the new blood vessels generated in the liver tumors (Fig. 7b). Contrast-enhanced axial images of the rabbit livers acquired at the total energy range and an iodine map image are shown in Fig. 7c,d, respectively. We observed a high signal at the edge of the liver tumor images. A comparison of the CNRs (mean ± SD) for tumors, livers, and aortas of the rabbits between those acquired from total energy range and from the iodine map is shown in Fig. 7e. As shown, the CNR of the iodine map is 1.7 to 2.3 times greater than that in the case of the total energy range.

### High-resolution imaging

**Figure 8:**
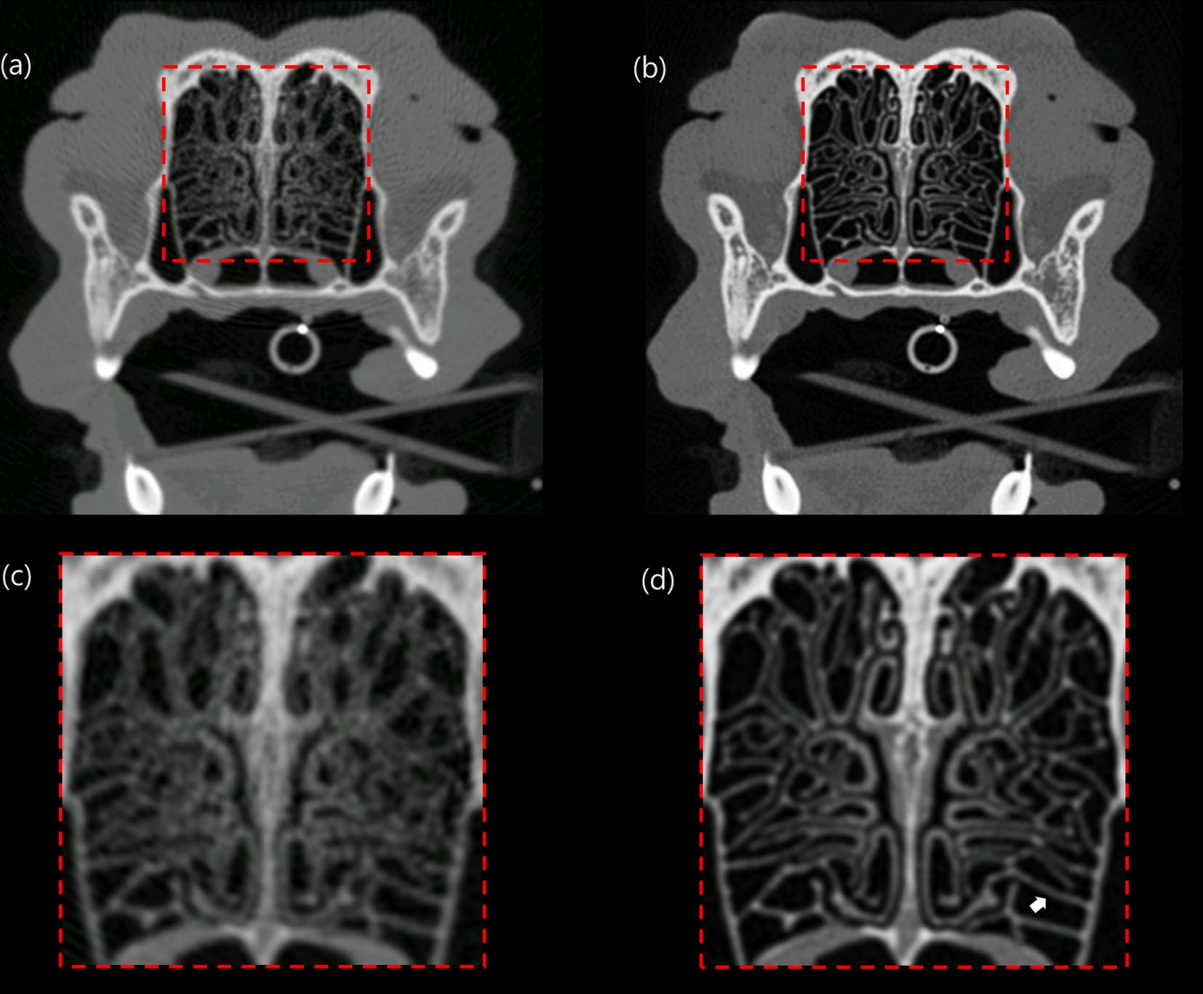
Axial images of the nasal turbinates scanned with the use of the (**a**) standard mode and (**b**) high-resolution (HR) mode of the PCD CT system. The average thickness of nasal turbinates (white arrow) is 0.5 mm. (**c**, **d**) The zoomed-in ROIs show better delineation of sub-millimeter nasal turbinates using (**c**) the HR modecompared with (**d**) the standard mode.

Figure 8 shows images of the thin turbinates in the dog's nose scanned with the use of a standard mode (a) and HR (b) mode of the PCD CT system. The magnified ROIs (c, d) show better delineation of sub-millimeter nasal turbinates with the use of the HR mode compared with the standard mode.

## Discussion

**Figure 9:**
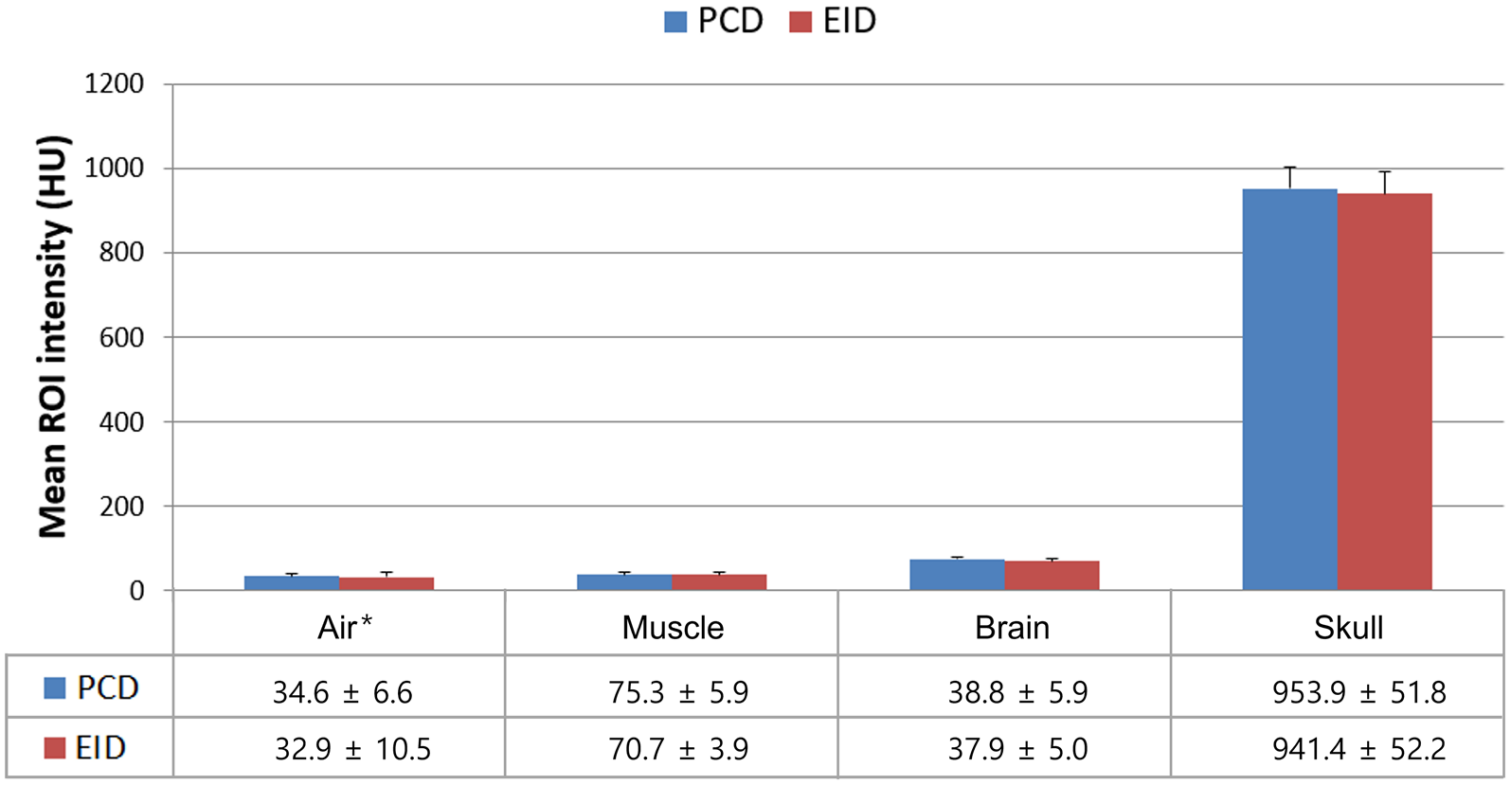
Mean HU values of air, muscle, brain, and skull image acquired from PCD and EID in Fig. 5. *The mean HU unit values for air were added to the value of 1000 HU for easier visualization.

In this study we investigated the usability of a prototypal CT system equipped with a PCD detector developed in-house for pre-clinical case studies. In addition, a deep-learning-based material decomposition algorithm was proposed and used in multi-material decomposition tasks. The study demonstrated several clinical imaging scenarios in the brain, liver, aorta, and nasal regions in two different animal models: a canine (head region) and a rabbit (liver with tumor, aorta). The pre- and post-contrast axial images of the canine’s brain reconstructed by the PCD and the EID CT systems were visually similar (Fig. 5), and the HU values of the materials in each tissue and the phantom also showed similar quantitative performances (Fig. 9). As shown in Fig. 6, the PCD CT system can acquire multi-energy images in a single scan, and use these multi-energy images to perform material decomposition and soft tissue segmentation^26^. CT imaging has been partially neglected for clinical diagnosis in the past due to its limited soft-tissue contrast characteristics. In particular, CT imaging has performed poorly in diagnosis of the acute phase of the ischemic stroke. Conversely, magnetic resonance perfusion^27^ and diffusion-weighted^28^ and FLAIR MRI^29,30^ have enabled such diagnoses.

After the brain tissue segmentation method using CT images proposed by DeLeo et al. in 1985^31^, various methods have been proposed for the same, for example,the heuristic rule-based method with adaptive intensity thresholding^32^, the method that uses probabilistic partial volume tissue maps based on a database of high-resolution longitudinal relaxation MRI^33^, and the weighted temporal average method^13^. However, precise brain tissue decomposition for the diagnosis of the acute phase of ischemic stroke has not been used in clinical practice yet. In this study, multi-energy images were acquired using a PCD CT system, and machine-learning-based material decomposition was performed to segment the white and gray matters for the first time. Further development of the methodology is required for case expansion, quantitative evaluation, improved accuracy and robustness. However, these initial brain tissue segmentation results, acquired from multi-energy images and the application of machine learning material decomposition, were important for subsequent applications, such as visualisation, brain volume measurements, automated detection, and quantification of cerebral pathology.

The model used for machine learning is still in its early stage of development. An optimised and lightweight version of the model is thus needed. We plan to conduct a study to optimise the number of hidden layers, weight size, and number of epochs. Furthermore, the current number of training data units is considerably small (16,875), and the model should be updated by increasing the number of training data to increase the accuracy. Training and testing of the current model were conducted with the use of individual pixel data. However, in the future, a study on accuracy improvement, optimisation, and the design of a lightweight version should be conducted based on the comparisons with well-known segmentation models.

The CNR values of each tissue were significantly higher in the iodine map than in the total energy range. In particular, the tumor-to-liver CNR was two times higher in the iodine map than in the total energy range, which suggested that the iodine map might serve as a practical technique for the detection of small hepatic and cerebrovascular tumors or residual and recurrent tumors after treatment. However, the ROI selected and analysed only hotspots of the tumor rather than the entire tumor, which could have affected the results^34^.

The HR mode was implemented in the PCD CT system in which the effective size of the z-axis pixel in the isocenter was 0.128 mm. In this study, the HR mode improved the spatial resolution and delineation of the fine structure in the dog nasal turbinates compared with the standard mode, thus showing the potential advantages of high-resolution images. Clinical applications that rely on high-spatial resolution such as the temporal bone, microvasculature, lung, and musculoskeletal CT imaging, are also likely to benefit from this resolution-enhancing function. However, at the time of the experiment, the data acquisition speed of the HR mode (with the use of 1 × 1 pixels) was too slow for clinical applications. Thus, the data transmission step has to be improved in the near future. After the evaluation of the detector performance and phantom by the multi-energy CT system developed based on the PCD detector^21^, animal images were acquired for the first time, and material separation and high-resolution performances were evaluated. However, the major limitation of this study was the small number of animals that were used. This limited the verification and statistical power of the results. Thus, additional studies are needed that consider a larger number of animals. Clinical PCD CT technology is still in the early stages of development, and many aspects, such as material decomposition, calibration, pre-processing, and the artifact reduction algorithm, which have been developed for the existing EID CT, would need to be optimised for the PCD CT system.

## Conclusions

In this study, preclinical experiments were performed using a prototypal PCD CT system developed in-house to compare its image quality and assess its material separation capability and high-resolution characteristics. The results demonstrated the potential impact of the HR images, the potential of the PCD CT system to enable improved detection of liver tumors, the increase of the utility of CT segmentation for brain imaging studies, and the capacity to acquire preclinical and clinical images.
